# Supplementary material for: Accelerating Chloroplast Engineering: A New System for Rapid Generation of Marker-Free Transplastomic Lines of Chlamydomonas reinhardtii
Source: Microorganisms. 2023 Jul 31;11(8):1967. doi: 10.3390/microorganisms11081967 (PMC10457852; doi:10.3390/microorganisms11081967)
Supplement: Supplementary file 1 [file microorganisms-11-01967-s001.zip › microorganisms-2468187-supplementary.pdf]

Supplementary data

Accelerating chloroplast engineering: a new system for rapid generation of marker-free transplastomic lines of *Chlamydomonas reinhardtii*

Henry N. Taunt*, Harry O. Jackson, Ísarr N. Gunnarsson, Rabbia Pervaiz, Saul Purton

Figure S1. DNA sequence of plasmids pHT158, pHT161 and pHT191 in Genbank format

Plasmid pHT158

```
LOCUS pHT158.psaA.5'Rest.     6166 bp ds-DNA     circular     27-FEB-2023
DEFINITION
FEATURES             Location/Qualifiers
     misc_feature    1..258
                     /label="rbcL 3'UTR-short"
     misc_feature    262..1072
                     /label="psaA 5' RF"
     misc_feature    1090..1176
                     /label="rrnB T1 terminator"
     misc_feature    1275..1379
                     /label="AmpR promoter"
     misc_feature    1380..2240
                     /label="AmpR"
     rep_origin      2335..3017
                     /label="ColE1 origin"
     misc_feature    3157..3251
                     /label="lambda t0 terminator"
     gene            complement(3263..4208)
                     /label="psaA exon 3"
     mRNA            3263..4663
                     /label="psaA 5' LF"
     group_II_intron complement(4209..4663)
                     /label="group_II_intron"
     misc_feature    4773..5368
                     /label="atpA promoter/5' UTR"
     misc_feature    5372..6163
                     /label="aadA CDS"
ORIGIN
        1 tttttatttt tcatgatgtt tatgtgaata gcataaacat cgtttttatt tttatggtgt
       61 ttaggttaaa tacctaaaca tcattttaca tttttaaaat taagttctaa agttatcttt
      121 tgtttaaatt tgcctgtctt tataaattac gatgtgccag aaaaataaaa tcttagcttt
      181 ttattataga atttatcttt atgtattata ttttataagt tataataaaa gaaatagtaa
      241 catactaaag cggatgtaGG AtactCAGct aactgccggg tagccttaaa gggtgaatgt
      301 atatacctag gcagttagcg gtgcctgtta aaatgcctcc ggcatccctg acgggacgct
      361 agtgaacgcc agtggcagtg gtacaataaa taaattgtat taaaatatta atataggacg
      421 tccccttacg ggacgccagt ggacgtcccc ttacgggacg ccagtggacg tccccttacg
      481 ggacgccagt ggacgtcccc ttacgggacg ccagtggacg tccccttacg ggacgccagt
      541 ggacgtcccc ttacgggacg ccagtggacg tccccttacg ggacgccagt ggacgtcccc
      601 ttacgggaat ataaatatta gtggacgtca gtggcagttg cctgccaact gcctatattt
      661 atatactccg aagtttactt gcctaggcag ttggcaggca actgccactg acgtccttcg
      721 gagtatataa atatcccaat ttttctatac ccgaagggga aggatataaa tatacttcgg
      781 agtattaaaa tatcggcagt tggcaggacg tccccttcgg gcaagtaaac ttcggagtat
      841 gtaaacattc tatatttata taccgctttt tgcattgccg cccgggctta attttatgcg
      901 actcctcgcc tttttatggc ctgcttcgct ggtacacggg caggtgaggt tttggtggcg
      961 gaatcactcc gttcttcttg ggagtgattc cgccaggacg ctttcgttgc cttctgggct
```

1021 cagttttgtt tggccatccg ccctttgtat ggcctgcttc gctcgtacGG AAGACCTCTA
1081 GGGCGGCGGA TTTGTCCTAC TCAGGAGAGC GTTCACCGAC AAACAACAGA TAAAACGAAA
1141 GGCCCAGTCT TTCGACTGAG CCTTTCGTTT TATTTGATGC CCGAAAGGGC CTCGTGATAC
1201 GCCTATTTTT ATAGGTTAAT GTCATGATAA TAATGGTTTC TTAGACGTCA GGTGGCACTT
1261 TTCGGGGAAA TGTGCGCGGA ACCCCTATTT GTTTATTTTT CTAAATACAT TCAAATATGT
1321 ATCCGCTCAT GAGACAATAA CCCTGATAAA TGCTTCAATA ATATTGAAAA AGGAAGAGTA
1381 TGAGTATTCA ACATTTCCGT GTCGCCCTTA TTCCCTTTTT TGCGGCATTT TGCCTTCCTG
1441 TTTTTGCTCA CCCAGAAACG CTGGTGAAAG TAAAAGATGC TGAAGATCAG TTGGGTGCAC
1501 GAGTGGGTTA CATCGAACTG GATCTCAACA GCGGTAAGAT CCTTGAGAGT TTTCGCCCCG
1561 AAGAACGTTT TCCAATGATG AGCACTTTTA AAGTTCTGCT ATGTGGCGCG GTATTATCCC
1621 GTATTGACGC CGGGCAAGAG CAACTCGGTC GCCGCATACA CTATTCTCAG AATGACTTGG
1681 TTGAGTACTC ACCAGTCACA GAAAAGCATC TTACGGATGG CATGACAGTA AGAGAATTAT
1741 GCAGTGCTGC CATAACCATG AGTGATAACA CTGCGGCCAA CTTACTTCTG ACAACGATCG
1801 GAGGACCGAA GGAGCTAACC GCTTTTTTGC ACAACATGGG GGATCATGTA ACTCGCCTTG
1861 ATCGTTGGGA ACCGGAGCTG AATGAAGCCA TACCAAACGA CGAGCGTGAC ACCACGATGC
1921 CTGTAGCAAT GGCAACAACG TTGCGCAAAC TATTAACTGG CGAACTACTT ACTCTAGCTT
1981 CCCGGCAACA ATTAATAGAC TGGATGGAGG CGGATAAAGT TGCAGGACCA CTTCTGCGCT
2041 CGGCCCTTCC GGCTGGCTGG TTTATTGCTG ATAAATCTGG AGCCGGTGAG CGTGGGTCCC
2101 GCGGTATCAT TGCAGCACTG GGGCCAGATG GTAAGCCCTC CCGTATCGTA GTTATCTACA
2161 CGACGGGGAG TCAGGCAACT ATGGATGAAC GAAATAGACA GATCGCTGAG ATAGGTGCCT
2221 CACTGATTAA GCATTGGTAA CTGTCAGACC AAGTTTACTC ATATATACTT TAGATTGATT
2281 TAAAACTTCA TTTTTAATTT AAAAGGATCT AGGTGAAGAT CCTTTTTGAT AATCTCATGA
2341 CCAAAATCCC TTAACGTGAG TTTTCGTTCC ACTGAGCGTC AGACCCCGTA GAAAAGATCA
2401 AAGGATCTTC TTGAGATCCT TTTTTTCTGC GCGTAATCTG CTGCTTGCAA ACAAAAAAAC
2461 CACCGCTACC AGCGGTGGTT TGTTTGCCGG ATCAAGAGCT ACCAACTCTT TTTCCGAAGG
2521 TAACTGGCTT CAGCAGAGCG CAGATACCAA ATACTGTTCT TCTAGTGTAG CCGTAGTTAG
2581 GCCACCACTT CAAGAACTCT GTAGCACCGC CTACATACCT CGCTCTGCTA ATCCTGTTAC
2641 CAGTGGCTGC TGCCAGTGGC GATAAGTCGT GTCTTACCGG GTTGGACTCA AGACGATAGT
2701 TACCGGATAA GGCGCAGCGG TCGGGCTGAA CGGGGGGTTC GTGCACACAG CCCAGCTTGG
2761 AGCGAACGAC CTACACCGAA CTGAGATACC TACAGCGTGA GCTATGAGAA AGCGCCACGC
2821 TTCCCGAAGG GAGAAAGGCG GACAGGTATC CGGTAAGCGG CAGGGTCGGA ACAGGAGAGC
2881 GCACGAGGGA GCTTCCAGGG GGAAACGCCT GGTATCTTTA TAGTCCTGTC GGGTTTCGCC
2941 ACCTCTGACT TGAGCGTCGA TTTTTGTGAT GCTCGTCAGG GGGGCGGAGC CTATGGAAAA
3001 ACGCCAGCAA CGCGGCCTTT TTACGGTTCC TGGCCTTTTG CTGGCCTTTT GCTCACATGT
3061 TCTTTCCTGC GTTATCCCCT GATTCTGTGG ATAACCGTAT TACCGCCTTT GAGTGAGCTG
3121 ATACCGCTCG CCGCAGCCGA ACGACCGAGC GCCTTGGACT CCTGTTGATA GATCCAGTAA
3181 TGACCTCAGA ACTCCATCTG GATTTGTTCA GAACGCTCGG TTGCCGCCGG GCGTTTTTTA
3241 TTGGTGAGAA TCCAGtgtgC AGagcaccaa caatacagaa accaccaatc catgtgtggt
3301 gtgtaaataa tgataattgt gtaccgtaat cagtagctaa ataagggtat ggaggcattg
3361 cgtacatgtg gtgagctaca ataattgata acgaaccaaa taaagctaag ttaatagcta
3421 attgtgcatg ccaagaagtt gttaaaattt catataaacc aacgtgacct tcacctgtaa
3481 atggaccacg gtgagcttct aaaatttctt tcatactgtg accaataccc cagttagtac
3541 gatacatgtg accagctact aagaataata cagcaatagc tacgtggtgg tgagcagtat
3601 cacttaacca aagaccacca gtaacagggt ttaaaccacc tttaaatgtt aagaaatcac
3661 tgtattcact ccagtttaaa gtaaagaaag gagcaatacc tttagcaaaa cttgggtata
3721 agtcagccat aatagcacga tttaataata aatcatgagg aagtggaatt tcttttggat
3781 ctacaccagc atctaataat ttgtttactg gtaaagaaac gtgaatttgg tgaccagccc
3841 aagctaaact acctaaacca agaagaccac ctaagtggtg gtttaacatt gattcaacgt
3901 tttggaacca ttctagtttt ggagcagctt tgtggtagtg gaaccaacca gcaaagaaca
3961 ttgcagcagc cattactaaa ccaccaattg ctgtagtata aagttgtaat tcactagtaa
4021 taccactagc acgccataat tggaagaaac cagaagtaat ttgaatacct tggaaaccac
4081 cacctacatc accgtttaaa atttcttgac ctacaatagg ccatactact tgagcacttg
4141 gtttaatgtg agtagggtca cttaaccaag cttcatagtt tgaaaaacgt gcaccatgga
4201 agtacatagt taggagttaa attttcttct ttaaaaattt atttaaacct taagagccgt
4261 gcatgcaatt ttcactgcac acggctcaaa ttctttctaa tgatttaatc caaggtagca
4321 gatttaacgc tatgcttgtt tataattatc tgtgttaaaa gcgcatgtat ttagggtgcc
4381 atttgctgcc atgcctcccc agagaggaag tttgtttatg ttaactcgcg cagtttgttt
4441 atgttaactc gcttggaaag tcatgctatt tatatacaat aaataaattt gtctcctcct
4501 ttccccttcc gggcaagggg gacatacttt ccaactacgt gccggcgtcc tgacggagcg
4561 tgtaaacctg ctcgtgccat aaataaaaaa tatcctatag ggatttgacg caagtttttg
4621 ctgtttttaa ataaatgtgt tatgcgtaat attatatgtt tttaaataaa tgtgttatgc

```
     4681 gtaatattat atatttacaa aatatatgat ctataaaata atttataaaa tatgccaata
     4741 ttttatggaa cgtaagccgt aaGGAGGAGC AGACGCGTCT CCAATATAGT AGACTTTATT
     4801 AGAGGCAGTG TTTATATACC ATAAACGTCA AAAGTCATTT TTATAACTGG ATCTCAAAAT
     4861 ACCTATAAAC CCATTGTTCT TCTCTTTTAG CTCTAAGAAC AATCAATTTA TAAATATATT
     4921 TATTATTATG CTATAATATA AATACTATAT AAATACATTT ACCTTTTTAT AAATACATTT
     4981 ACCTTTTTTT TAATTTGCAT GATTTTAATG CTTATGCTAT CTTTTTTATT TAGTCCATAA
     5041 AACCTTTAAA GGACCTTTTC TTATGGGATA TTTATATTTT CCTAACAAAG CAATCGGCGT
     5101 CATAAACTTT AGTTGCTTAC GACGCCTGTG GACGTCCCCC CCTTCCCCTT ACGGGCAAGT
     5161 AAACTTAGGG ATTTTAATGC AATAAATAAA TTTGTCCTCT TCGGGCAAAT GAATTTTAGT
     5221 ATTTAAATAT GACAAGGGTG AACCATTACT TTTGTTAACA AGTGATCTTA CCACTCACTA
     5281 TTTTTGTTGA ATTTTAAACT TATTTAAAAT TCTCGAGAAA GATTTTAAAA ATAAACTTTT
     5341 TTAATCTTTT ATTTATTTTT TCTTTTTTAT GGCTCGTGAA GCGGTTATCG CCGAAGTATC
     5401 AACTCAACTA TCAGAGGTAG TTGGCGTCAT CGAGCGCCAT CTCGAACCGA CGTTGCTGGC
     5461 CGTACATTTG TACGGCTCCG CAGTGGATGG CGGCCTGAAG CCACACAGTG ATATTGATTT
     5521 GCTGGTTACG GTGACCGTAA GGCTTGATGA AACAACGCGG CGAGCTTTGA TCAACGACCT
     5581 TTTGGAAACT TCGGCTTCCC CTGGAGAGAG CGAGATTCTC CGCGCTGTAG AAGTCACCAT
     5641 TGTTGTGCAC GACGACATCA TTCCGTGGCG TTATCCAGCT AAGCGCGAAC TGCAATTTGG
     5701 AGAATGGCAG CGCAATGACA TTCTTGCAGG TATCTTCGAG CCAGCCACGA TCGACATTGA
     5761 TCTGGCTATC TTGCTGACAA AAGCAAGAGA ACATAGCGTT GCCTTGGTAG GTCCAGCGGC
     5821 GGAGGAACTC TTTGATCCGG TTCCTGAACA GGATCTATTT GAGGCGCTAA ATGAAACCTT
     5881 AACGCTATGG AACTCGCCGC CCGACTGGGC TGGCGATGAG CGAAATGTAG TGCTTACGTT
     5941 GTCCCGCATT TGGTACAGCG CAGTAACCGG CAAAATCGCG CCGAAGGATG TCGCTGCCGA
     6001 CTGGGCAATG GAGCGCCTGC CGGCCCAGTA TCAGCCCGTC ATACTTGAAG CTAGACAGGC
     6061 TTATCTTGGA CAAGAAGAAG ATCGCTTGGC CTCGCGCGCA GATCAGTTGG AAGAATTTGT
     6121 CCACTACGTG AAAGGCGAGA TCACTAAGGT AGTTGGCAAA TAATAA
//
```

Plasmid pHT161

```
LOCUS pHT161.psaA.5'.KO       5466 bp ds-DNA     circular     27-FEB-2023
DEFINITION
FEATURES             Location/Qualifiers
     misc_feature    1..596
                     /label="atpA promoter/5' UTR"
     misc_feature    600..1391
                     /label="aadA CDS"
     misc_feature    1395..1652
                     /label="rbcL 3'UTR-short"
     misc_feature    1656..2466
                     /label="psaA 5' RF"
     misc_feature    2484..2570
                     /label="rrnB T1 terminator"
     misc_feature    2669..2773
                     /label="AmpR promoter"
     misc_feature    2774..3634
                     /label="AmpR"
     rep_origin      3729..4411
                     /label="ColE1 origin"
     misc_feature    4551..4645
                     /label="lambda t0 terminator"
     gene            4657..5456
                     /label="psa 5' KO LF"
ORIGIN
        1 ACGCGTCTCC AATATAGTAG ACTTTATTAG AGGCAGTGTT TATATACCAT AAACGTCAAA
       61 AGTCATTTTT ATAACTGGAT CTCAAAATAC CTATAAACCC ATTGTTCTTC TCTTTTAGCT
      121 CTAAGAACAA TCAATTTATA AATATATTTA TTATTATGCT ATAATATAAA TACTATATAA
      181 ATACATTTAC CTTTTTATAA ATACATTTAC CTTTTTTTTA ATTTGCATGA TTTTAATGCT
      241 TATGCTATCT TTTTTATTTA GTCCATAAAA CCTTTAAAGG ACCTTTTCTT ATGGGATATT
      301 TATATTTTCC TAACAAAGCA ATCGGCGTCA TAAACTTTAG TTGCTTACGA CGCCTGTGGA
      361 CGTCCCCCCC TTCCCCTTAC GGGCAAGTAA ACTTAGGGAT TTTAATGCAA TAAATAAATT
      421 TGTCCTCTTC GGGCAAATGA ATTTTAGTAT TTAAATATGA CAAGGGTGAA CCATTACTTT
```

481 TGTTAACAAG TGATCTTACC ACTCACTATT TTTGTTGAAT TTTAAACTTA TTTAAAATTC
541 TCGAGAAAGA TTTTAAAAAT AAACTTTTTT AATCTTTTAT TTATTTTTTC TTTTTTATGG
601 CTCGTGAAGC GGTTATCGCC GAAGTATCAA CTCAACTATC AGAGGTAGTT GGCGTCATCG
661 AGCGCCATCT CGAACCGACG TTGCTGGCCG TACATTTGTA CGGCTCCGCA GTGGATGGCG
721 GCCTGAAGCC ACACAGTGAT ATTGATTTGC TGGTTACGGT GACCGTAAGG CTTGATGAAA
781 CAACGCGGCG AGCTTTGATC AACGACCTTT TGGAAACTTC GGCTTCCCCT GGAGAGAGCG
841 AGATTCTCCG CGCTGTAGAA GTCACCATTG TTGTGCACGA CGACATCATT CCGTGGCGTT
901 ATCCAGCTAA GCGCGAACTG CAATTTGGAG AATGGCAGCG CAATGACATT CTTGCAGGTA
961 TCTTCGAGCC AGCCACGATC GACATTGATC TGGCTATCTT GCTGACAAAA GCAAGAGAAC
1021 ATAGCGTTGC CTTGGTAGGT CCAGCGGCGG AGGAACTCTT TGATCCGGTT CCTGAACAGG
1081 ATCTATTTGA GGCGCTAAAT GAAACCTTAA CGCTATGGAA CTCGCCGCCC GACTGGGCTG
1141 GCGATGAGCG AAATGTAGTG CTTACGTTGT CCCGCATTTG GTACAGCGCA GTAACCGGCA
1201 AAATCGCGCC GAAGGATGTC GCTGCCGACT GGGCAATGGA GCGCCTGCCG GCCCAGTATC
1261 AGCCCGTCAT ACTTGAAGCT AGACAGGCTT ATCTTGGACA AGAAGAAGAT CGCTTGGCCT
1321 CGCGCGCAGA TCAGTTGGAA GAATTTGTCC ACTACGTGAA AGGCGAGATC ACTAAGGTAG
1381 TTGGCAAATA ATAAttttta tttttcatga tgtttatgtg aatagcataa acatcgtttt
1441 tatttttatg gtgtttaggt taaataccta aacatcattt tacattttta aaattaagtt
1501 ctaaagttat cttttgttta aatttgcctg tctttataaa ttacgatgtg ccagaaaaat
1561 aaaatcttag ctttttatta tagaatttat ctttatgtat tatattttat aagttataat
1621 aaaagaaata gtaacatact aaagcggatg taGGAtactC AGctaactgc cgggtagcct
1681 taaagggtga atgtatatac ctaggcagtt agcggtgcct gttaaaatgc ctccggcatc
1741 cctgacggga cgctagtgaa cgccagtggc agtggtacaa taaataaatt gtattaaaat
1801 attaatatag gacgtcccct tacgggacgc cagtggacgt ccccttacgg gacgccagtg
1861 gacgtcccct tacgggacgc cagtggacgt ccccttacgg gacgccagtg gacgtcccct
1921 tacgggacgc cagtggacgt ccccttacgg gacgccagtg gacgtcccct tacgggacgc
1981 cagtggacgt ccccttacgg gaatataaat attagtggac gtcagtggca gttgcctgcc
2041 aactgcctat atttatatac tccgaagttt acttgcctag gcagttggca ggcaactgcc
2101 actgacgtcc ttcggagtat ataaatatcc caatttttct atacccgaag ggaaggata
2161 taaatatact tcggagtatt aaaatatcgg cagttggcag gacgtcccct tcgggcaagt
2221 aaacttcgga gtatgtaaac attctatatt tatataccgc tttttgcatt gccgcccggg
2281 cttaatttta tgcgactcct cgccttttta tggcctgctt cgctggtaca cgggcaggtg
2341 aggttttggt ggcggaatca ctccgttctt cttgggagtg attccgccag gacgctttcg
2401 ttgccttctg ggctcagttt tgtttggcca tccgcccttt gtatggcctg cttcgctcgt
2461 acGGAAGACC TCTAGGGCGG CGGATTTGTC CTACTCAGGA GAGCGTTCAC CGACAAACAA
2521 CAGATAAAAC GAAAGGCCCA GTCTTTCGAC TGAGCCTTTC GTTTTATTTG ATGCCCGAAA
2581 GGGCCTCGTG ATACGCCTAT TTTTATAGGT TAATGTCATG ATAATAATGG TTTCTTAGAC
2641 GTCAGGTGGC ACTTTTCGGG GAAATGTGCG CGGAACCCCT ATTTGTTTAT TTTTCTAAAT
2701 ACATTCAAAT ATGTATCCGC TCATGAGACA ATAACCCTGA TAAATGCTTC AATAATATTG
2761 AAAAAGGAAG AGTATGAGTA TTCAACATTT CCGTGTCGCC CTTATTCCCT TTTTTGCGGC
2821 ATTTTGCCTT CCTGTTTTTG CTCACCCAGA AACGCTGGTG AAAGTAAAAG ATGCTGAAGA
2881 TCAGTTGGGT GCACGAGTGG GTTACATCGA ACTGGATCTC AACAGCGGTA AGATCCTTGA
2941 GAGTTTTCGC CCCGAAGAAC GTTTTCCAAT GATGAGCACT TTTAAAGTTC TGCTATGTGG
3001 CGCGGTATTA TCCCGTATTG ACGCCGGGCA AGAGCAACTC GGTCGCCGCA TACACTATTC
3061 TCAGAATGAC TTGGTTGAGT ACTCACCAGT CACAGAAAAG CATCTTACGG ATGGCATGAC
3121 AGTAAGAGAA TTATGCAGTG CTGCCATAAC CATGAGTGAT AACACTGCGG CCAACTTACT
3181 TCTGACAACG ATCGGAGGAC CGAAGGAGCT AACCGCTTTT TTGCACAACA TGGGGGATCA
3241 TGTAACTCGC CTTGATCGTT GGGAACCGGA GCTGAATGAA GCCATACCAA ACGACGAGCG
3301 TGACACCACG ATGCCTGTAG CAATGGCAAC AACGTTGCGC AAACTATTAA CTGGCGAACT
3361 ACTTACTCTA GCTTCCCGGC AACAATTAAT AGACTGGATG GAGGCGGATA AAGTTGCAGG
3421 ACCACTTCTG CGCTCGGCCC TTCCGGCTGG CTGGTTTATT GCTGATAAAT CTGGAGCCGG
3481 TGAGCGTGGG TCCCGCGGTA TCATTGCAGC ACTGGGGCCA GATGGTAAGC CCTCCCGTAT
3541 CGTAGTTATC TACACGACGG GGAGTCAGGC AACTATGGAT GAACGAAATA GACAGATCGC
3601 TGAGATAGGT GCCTCACTGA TTAAGCATTG GTAACTGTCA GACCAAGTTT ACTCATATAT
3661 ACTTTAGATT GATTTAAAAC TTCATTTTTA ATTTAAAAGG ATCTAGGTGA AGATCCTTTT
3721 TGATAATCTC ATGACCAAAA TCCCTTAACG TGAGTTTTCG TTCCACTGAG CGTCAGACCC
3781 CGTAGAAAAG ATCAAAGGAT CTTCTTGAGA TCCTTTTTTT CTGCGCGTAA TCTGCTGCTT
3841 GCAAACAAAA AAACCACCGC TACCAGCGGT GGTTTGTTTG CCGGATCAAG AGCTACCAAC
3901 TCTTTTTCCG AAGGTAACTG GCTTCAGCAG AGCGCAGATA CCAAATACTG TTCTTCTAGT
3961 GTAGCCGTAG TTAGGCCACC ACTTCAAGAA CTCTGTAGCA CCGCCTACAT ACCTCGCTCT
4021 GCTAATCCTG TTACCAGTGG CTGCTGCCAG TGGCGATAAG TCGTGTCTTA CCGGGTTGGA
4081 CTCAAGACGA TAGTTACCGG ATAAGGCGCA GCGGTCGGGC TGAACGGGGG GTTCGTGCAC

```
     4141 ACAGCCCAGC TTGGAGCGAA CGACCTACAC CGAACTGAGA TACCTACAGC GTGAGCTATG
     4201 AGAAAGCGCC ACGCTTCCCG AAGGGAGAAA GGCGGACAGG TATCCGGTAA GCGGCAGGGT
     4261 CGGAACAGGA GAGCGCACGA GGGAGCTTCC AGGGGGAAAC GCCTGGTATC TTTATAGTCC
     4321 TGTCGGGTTT CGCCACCTCT GACTTGAGCG TCGATTTTTG TGATGCTCGT CAGGGGGGCG
     4381 GAGCCTATGG AAAAACGCCA GCAACGCGGC CTTTTTACGG TTCCTGGCCT TTTGCTGGCC
     4441 TTTTGCTCAC ATGTTCTTTC CTGCGTTATC CCCTGATTCT GTGGATAACC GTATTACCGC
     4501 CTTTGAGTGA GCTGATACCG CTCGCCGCAG CCGAACGACC GAGCGCCTTG GACTCCTGTT
     4561 GATAGATCCA GTAATGACCT CAGAACTCCA TCTGGATTTG TTCAGAACGC TCGGTTGCCG
     4621 CCGGGCGTTT TTTATTGGTG AGAATCCAGt gtgCAGagca ccaacaatac agaaaccacc
     4681 aatccatgtg tggtgtgtaa ataatgataa ttgtgtaccg taatcagtag ctaaataagg
     4741 gtatggaggc attgcgtaca tgtggtgagc tacaataatt gataacgaac caaataaagc
     4801 taagttaata gctaattgtg catgccaaga agttgttaaa atttcatata aaccaacgtg
     4861 accttcacct gtaaatggac cacggtgagc ttctaaaatt tctttcatac tgtgaccaat
     4921 accccagtta gtacgataca tgtgaccagc tactaagaat aatacagcaa tagctacgtg
     4981 gtggtgagca gtatcactta accaaagacc accagtaaca gggtttaaac cacctttaaa
     5041 tgttaagaaa tcactgtatt cactccagtt taaagtaaag aaaggagcaa tacctttagc
     5101 aaaacttggg tataagtcag ccataatagc acgatttaat aataaatcat gaggaagtgg
     5161 aatttctttt ggatctacac cagcatctaa taatttgttt actggtaaag aaacgtgaat
     5221 ttggtgacca gcccaagcta aactacctaa accaagaaga ccacctaagt ggtggtttaa
     5281 cattgattca acgttttgga accattctag ttttggagca gctttgtggt agtggaacca
     5341 accagcaaag aacattgcag cagccattac taaaccacca attgctgtag tataaagttg
     5401 taattcacta gtaataccac tagcacgcca taattggaag aaaccagaag taatttGGAG
     5461 GAGCAG
//
```

Plasmid pHT191

```
LOCUS pHT191_Lv.2.rrnSp.psaA5 6772 bp ds-DNA     circular     27-FEB-2023
DEFINITION
FEATURES             Location/Qualifiers
     rep_origin      1..683
                     /label="ColE1 origin"
     misc_feature    823..917
                     /label="lambda t0 terminator"
     gene            complement(929..1874)
                     /label="psaA exon 3"
     mRNA            929..2329
                     /label="psaA 5' LF"
     group_II_intron complement(1875..2329)
                     /label="group_II_intron"
     misc_feature    2436..2438
                     /label="SapI fusion site"
     misc_feature    2439..2651
                     /label="16S rRNA promoter"
     misc_feature    2652..2785
                     /label="psaA 5'UTR"
     Gene            2786..4435
                     /label="LucCP"
     misc_feature    4439..4696
                     /label="rbcL 3'UTR-short"
     misc_feature    4700..5510
                     /label="psaA 5' RF"
     misc_feature    5528..5614
                     /label="rrnB T1 terminator"
     misc_feature    5713..5817
                     /label="AmpR promoter"
     misc_feature    5818..6678
                     /label="AmpR"
ORIGIN
        1 TCATGACCAA AATCCCTTAA CGTGAGTTTT CGTTCCACTG AGCGTCAGAC CCCGTAGAAA
       61 AGATCAAAGG ATCTTCTTGA GATCCTTTTT TTCTGCGCGT AATCTGCTGC TTGCAAACAA
```

121 AAAAACCACC GCTACCAGCG GTGGTTTGTT TGCCGGATCA AGAGCTACCA ACTCTTTTTC
181 CGAAGGTAAC TGGCTTCAGC AGAGCGCAGA TACCAAATAC TGTTCTTCTA GTGTAGCCGT
241 AGTTAGGCCA CCACTTCAAG AACTCTGTAG CACCGCCTAC ATACCTCGCT CTGCTAATCC
301 TGTTACCAGT GGCTGCTGCC AGTGGCGATA AGTCGTGTCT TACCGGGTTG GACTCAAGAC
361 GATAGTTACC GGATAAGGCG CAGCGGTCGG GCTGAACGGG GGGTTCGTGC ACACAGCCCA
421 GCTTGGAGCG AACGACCTAC ACCGAACTGA GATACCTACA GCGTGAGCTA TGAGAAAGCG
481 CCACGCTTCC CGAAGGGAGA AAGGCGGACA GGTATCCGGT AAGCGGCAGG GTCGGAACAG
541 GAGAGCGCAC GAGGGAGCTT CCAGGGGGAA ACGCCTGGTA TCTTTATAGT CCTGTCGGGT
601 TTCGCCACCT CTGACTTGAG CGTCGATTTT TGTGATGCTC GTCAGGGGGG CGGAGCCTAT
661 GGAAAAACGC CAGCAACGCG GCCTTTTTAC GGTTCCTGGC CTTTTGCTGG CCTTTTGCTC
721 ACATGTTCTT TCCTGCGTTA TCCCCTGATT CTGTGGATAA CCGTATTACC GCCTTTGAGT
781 GAGCTGATAC CGCTCGCCGC AGCCGAACGA CCGAGCGCCT TGGACTCCTG TTGATAGATC
841 CAGTAATGAC CTCAGAACTC CATCTGGATT TGTTCAGAAC GCTCGGTTGC CGCCGGGCGT
901 TTTTTATTGG TGAGAATCCA GtgtgCAGag caccaacaat acagaaacca ccaatccatg
961 tgtggtgtgt aaataatgat aattgtgtac cgtaatcagt agctaaataa gggtatggag
1021 gcattgcgta catgtggtga gctacaataa ttgataacga accaaataaa gctaagttaa
1081 tagctaattg tgcatgccaa gaagttgtta aaatttcata taaaccaacg tgaccttcac
1141 ctgtaaatgg accacggtga gcttctaaaa tttctttcat actgtgacca ataccccagt
1201 tagtacgata catgtgacca gctactaaga ataatacagc aatagctacg tggtggtgag
1261 cagtatcact taaccaaaga ccaccagtaa cagggtttaa accaccttta aatgttaaga
1321 aatcactgta ttcactccag tttaaagtaa agaaaggagc aataccttta gcaaaacttg
1381 ggtataagtc agccataata gcacgattta ataataaatc atgaggaagt ggaatttctt
1441 ttggatctac accagcatct aataatttgt ttactggtaa agaaacgtga atttggtgac
1501 cagcccaagc taaactacct aaaccaagaa gaccacctaa gtggtggttt aacattgatt
1561 caacgttttg gaaccattct agttttggag cagctttgtg gtagtggaac caaccagcaa
1621 agaacattgc agcagccatt actaaaccac caattgctgt agtataaagt tgtaattcac
1681 tagtaatacc actagcacgc cataattgga agaaaccaga agtaatttga ataccttgga
1741 aaccaccacc tacatcaccg tttaaaattt cttgacctac aataggccat actacttgag
1801 cacttggttt aatgtgagta gggtcactta accaagcttc atagtttgaa aaacgtgcac
1861 catggaagta catagttagg agttaaattt tcttctttaa aaatttattt aaaccttaag
1921 agccgtgcat gcaattttca ctgcacacgg ctcaaattct ttctaatgat ttaatccaag
1981 gtagcagatt taacgctatg cttgtttata attatctgtg ttaaaagcgc atgtatttag
2041 ggtgccattt gctgccatgc ctccccagag aggaagtttg tttatgttaa ctcgcgcagt
2101 ttgtttatgt taactcgctt ggaaagtcat gctatttata tacaataaat aaatttgtct
2161 cctcctttcc ccttccgggc aaggggggaca tactttccaa ctacgtgccg gcgtcctgac
2221 ggagcgtgta aacctgctcg tgccataaat aaaaaatatc ctatagggat ttgacgcaag
2281 tttttgctgt ttttaaataa atgtgttatg cgtaatatta tatgttttta aataaatgtg
2341 ttatgcgtaa tattatatat ttacaaaata tatgatctat aaaataattt ataaaatatg
2401 ccaatatttt atggaacgta agccgtaaGG AGGAGCAGGG CAGGCAACAA ATTTATTTAT
2461 TGTCCCGTAA GGGGAAGGGG AAAACAATTA TTATTTTACT GCGGAGCAGC TTGTTATTGA
2521 AATTTTATTA AAAAAAAAAT AAAAATTTGA CAAAAAAAAA TAAAAAAGTT AAATTAAAAA
2581 CACTGGGAAT GTTCTACATC ATAAAAATCA AAAGGGTTTA AAATCCCGAC AAAATTTAAA
2641 CTTTAAAGAG TATGATGTAA AAAAAACTAT TTGTCTAATT TAATAACCAT GCATTTTTTA
2701 TGAACACATA ATAATTAAAA GCGTTGCTAA TGGTGTAAAT AATGTATTTA TTAAATTAAA
2761 TAATTGTTAT TATAAGGAGA AATCCATGGA AGATGCTAAA AATATTAAAA AAGGTCCAGC
2821 TCCATTTTAT CCATTAGAAG ATGGTACAGC TGGTGAACAA TTACATAAAG CTATGAAACG
2881 TTATGCTTTA GTTCCAGGTA CAATTGCTTT TACAGATGCT CATATTGAAG TTGATATTAC
2941 ATATGCTGAA TATTTTGAAA TGTCAGTTCG TTTAGCTGAA GCTATGAAAC GTTATGGTTT
3001 AAATACAAAT CATCGTATTG TTGTTTGTTC AGAAAATTCA TTACAATTTT TTATGCCAGT
3061 TTTAGGTGCT TTATTTATTG GTGTTGCTGT TGCTCCAGCT AATGATATTT ATAATGAACG
3121 TGAATTATTA AATTCAATGG GTATTTCACA ACCAACAGTT GTTTTTGTTT CAAAAAAAGG
3181 TTTACAAAAA ATTTTAAATG TTCAAAAAAA ATTACCAATT ATTCAAAAAA TTATTATTAT
3241 GGATTCAAAA ACAGATTATC AAGGTTTTCA ATCAATGTAT ACATTTGTTA CATCACATTT
3301 ACCACCAGGT TTTAATGAAT ATGATTTTGT TCCAGAATCA TTTGATCGTG ATAAAACAAT
3361 TGCTTTAATT ATGAATTCAT CAGGTTCAAC AGGTTTACCA AAAGGTGTTG CTTTACCACA
3421 TCGTACAGCT TGTGTTCGTT TTTCACATGC TCGTGATCCA ATTTTTGGTA ATCAAATTAT
3481 TCCAGATACA GCTATTTTAT CAGTTGTTCC ATTTCATCAT GGTTTTGGTA TGTTTACAAC
3541 ATTAGGTTAT TTAATTTGTG GTTTTCGTGT TGTTTTAATG TATCGTTTTG AAGAAGAATT
3601 ATTTTTACGT TCATTACAAG ATTATAAAAT TCAATCAGCT TTATTAGTTC CAACATTATT
3661 TTCATTTTTT GCTAAATCAA CATTAATTGA TAAATATGAT TTATCAAATT TACATGAAAT
3721 TGCTTCAGGT GGTGCTCCAT TATCAAAAGA AGTTGGTGAA GCTGTTGCTA AACGTTTTCA

3781 TTTACCAGGT ATTCGTCAAG GTTATGGTTT AACAGAAACA ACATCAGCTA TTTTAATTAC
3841 ACCAGAAGGT GATGATAAAC CAGGTGCTGT TGGTAAAGTT GTTCCATTTT TTGAAGCTAA
3901 AGTTGTTGAT TTAGATACAG GTAAAACATT AGGTGTTAAT CAACGTGGTG AATTATGTGT
3961 TCGTGGTCCA ATGATTATGT CAGGTTATGT TAATAATCCA GAAGCTACAA ATGCTTTAAT
4021 TGATAAAGAT GGTTGGTTAC ATTCAGGTGA TATTGCTTAT TGGGATGAAG ATGAACATTT
4081 TTTTATTGTT GATCGTTTAA AATCATTAAT TAAATATAAA GGTTATCAAG TTGCTCCAGC
4141 TGAATTAGAA TCAATTTTAT TACAACATCC AAATATTTTT GATGCTGGTG TTGCTGGTTT
4201 ACCAGATGAT GATGCTGGTG AATTACCAGC TGCTGTTGTT GTTTTAGAAC ATGGTAAAAC
4261 AATGACAGAA AAAGAAATTG TTGATTATGT TGCTTCACAA GTTACAACAG CTAAAAAATT
4321 ACGTGGTGGT GTTGTTTTTG TTGATGAAGT TCCAAAAGGT TTAACAGGTA AATTAGATGC
4381 TCGTAAAATT CGTGAAATTT TAATTAAAGC TAAAAAAGGT GGTAAAATTG CTGTTTAAtt
4441 tttatttttc atgatgttta tgtgaatagc ataaacatcg tttttatttt tatggtgttt
4501 aggttaaata cctaaacatc atttacatt tttaaaatta agttctaaag ttatcttttg
4561 tttaaatttg cctgtcttta taaattacga tgtgccagaa aaataaaatc ttagcttttt
4621 attatagaat ttatctttat gtattatatt ttataagtta taataaaaga aatagtaaca
4681 tactaaagcg gatgtaGGAt actCAGctaa ctgccgggta gccttaaagg gtgaatgtat
4741 atacctaggc agttagcggt gcctgttaaa atgcctccgg catccctgac gggacgctag
4801 tgaacgccag tggcagtggt acaataaata aattgtatta aaatattaat ataggacgtc
4861 cccttacggg acgccagtgg acgtcccctt acgggacgcc agtggacgtc cccttacggg
4921 acgccagtgg acgtcccctt acgggacgcc agtggacgtc cccttacggg acgccagtgg
4981 acgtcccctt acgggacgcc agtggacgtc cccttacggg acgccagtgg acgtcccctt
5041 acgggaatat aaatattagt ggacgtcagt ggcagttgcc tgccaactgc ctatatttat
5101 atactccgaa gtttacttgc ctaggcagtt ggcaggcaac tgccactgac gtccttcgga
5161 gtatataaat atcccaattt ttctataccc gaaggggaag gatataaata tacttcggag
5221 tattaaaata tcggcagttg gcaggacgtc cccttcgggc aagtaaactt cggagtatgt
5281 aaacattcta tatttatata ccgctttttg cattgccgcc cgggcttaat tttatgcgac
5341 tcctcgcctt tttatggcct gcttcgctgg tacacgggca ggtgaggttt tggtggcgga
5401 atcactccgt tcttcttggg agtgattccg ccaggacgct ttcgttgcct tctgggctca
5461 gttttgtttg gccatccgcc ctttgtatgg cctgcttcgc tcgtacGGAA GACCTCTAGG
5521 GCGGCGGATT TGTCCTACTC AGGAGAGCGT TCACCGACAA ACAACAGATA AAACGAAAGG
5581 CCCAGTCTTT CGACTGAGCC TTTCGTTTTA TTTGATGCCC GAAAGGGCCT CGTGATACGC
5641 CTATTTTTAT AGGTTAATGT CATGATAATA ATGGTTTCTT AGACGTCAGG TGGCACTTTT
5701 CGGGGAAATG TGCGCGGAAC CCCTATTTGT TTATTTTTCT AAATACATTC AAATATGTAT
5761 CCGCTCATGA GACAATAACC CTGATAAATG CTTCAATAAT ATTGAAAAAG GAAGAGTATG
5821 AGTATTCAAC ATTTCCGTGT CGCCCTTATT CCCTTTTTTG CGGCATTTTG CCTTCCTGTT
5881 TTTGCTCACC CAGAAACGCT GGTGAAAGTA AAAGATGCTG AAGATCAGTT GGGTGCACGA
5941 GTGGGTTACA TCGAACTGGA TCTCAACAGC GGTAAGATCC TTGAGAGTTT TCGCCCCGAA
6001 GAACGTTTTC CAATGATGAG CACTTTTAAA GTTCTGCTAT GTGGCGCGGT ATTATCCCGT
6061 ATTGACGCCG GGCAAGAGCA ACTCGGTCGC CGCATACACT ATTCTCAGAA TGACTTGGTT
6121 GAGTACTCAC CAGTCACAGA AAAGCATCTT ACGGATGGCA TGACAGTAAG AGAATTATGC
6181 AGTGCTGCCA TAACCATGAG TGATAACACT GCGGCCAACT TACTTCTGAC AACGATCGGA
6241 GGACCGAAGG AGCTAACCGC TTTTTTGCAC AACATGGGGG ATCATGTAAC TCGCCTTGAT
6301 CGTTGGGAAC CGGAGCTGAA TGAAGCCATA CCAAACGACG AGCGTGACAC CACGATGCCT
6361 GTAGCAATGG CAACAACGTT GCGCAAACTA TTAACTGGCG AACTACTTAC TCTAGCTTCC
6421 CGGCAACAAT TAATAGACTG GATGGAGGCG GATAAAGTTG CAGGACCACT TCTGCGCTCG
6481 GCCCTTCCGG CTGGCTGGTT TATTGCTGAT AAATCTGGAG CCGGTGAGCG TGGGTCCCGC
6541 GGTATCATTG CAGCACTGGG GCCAGATGGT AAGCCCTCCC GTATCGTAGT TATCTACACG
6601 ACGGGGAGTC AGGCAACTAT GGATGAACGA AATAGACAGA TCGCTGAGAT AGGTGCCTCA
6661 CTGATTAAGC ATTGGTAACT GTCAGACCAA GTTTACTCAT ATATACTTTA GATTGATTTA
6721 AAACTTCATT TTTAATTTAA AAGGATCTAG GTGAAGATCC TTTTTGATAA TC
//

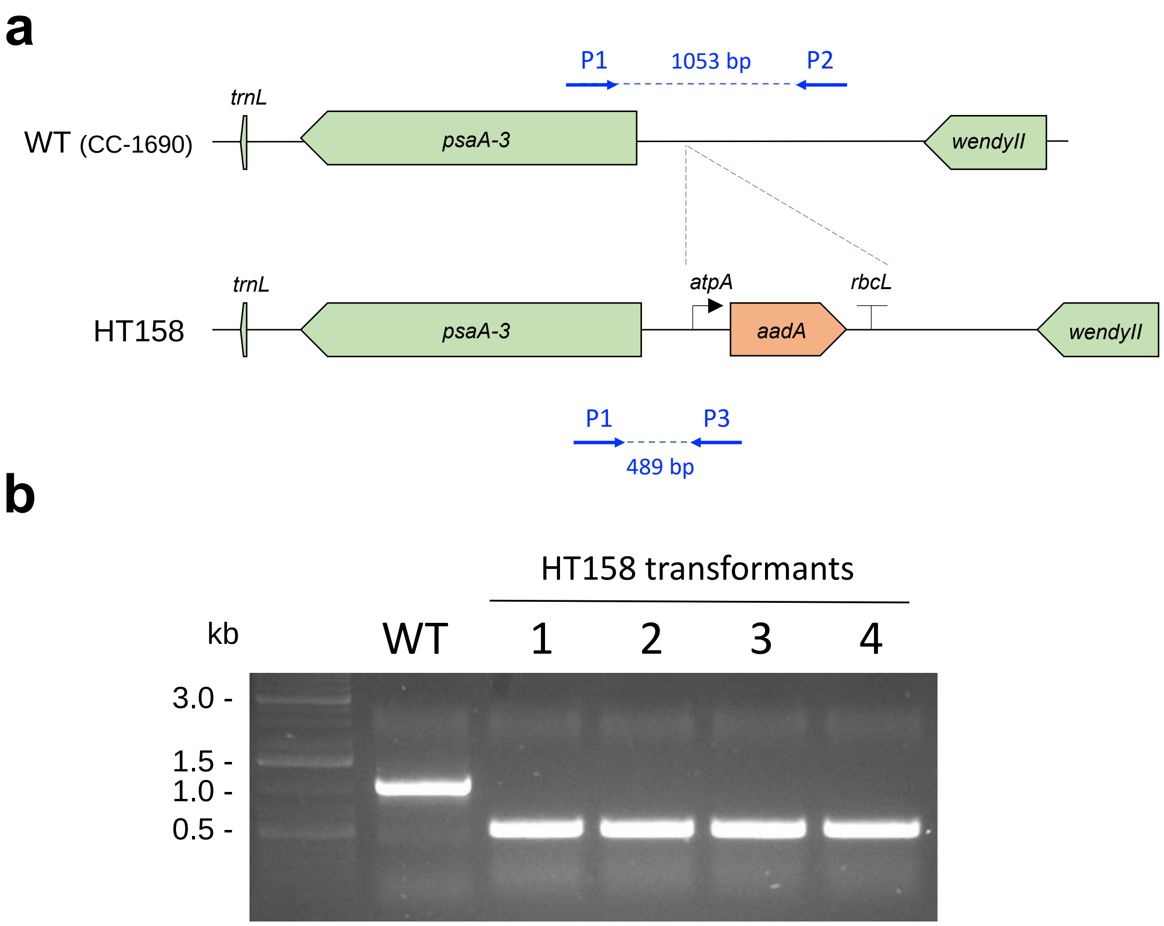

Figure S2. Confirmation of homoplasmy in HT158 transformants. Total genomic DNA isolated from the untransformed wild-type (WT) strain CC-1690 and from four independent transformant lines was used for PCR analysis of the *psaA-3* locus. A three-primer reaction allowed for discrimination of the two alleles with primers 1+2 specific for the WT allele and giving a PCR product of 1053 bp, and primers 1+3 specific for the allele containing the *aadA* cassette and giving a product of 489 bp. The absence of a WT band in the HT158 analysis indicates that the strains are homoplasmic (*i.e.* contains only transformed plastome copies within its chloroplast).

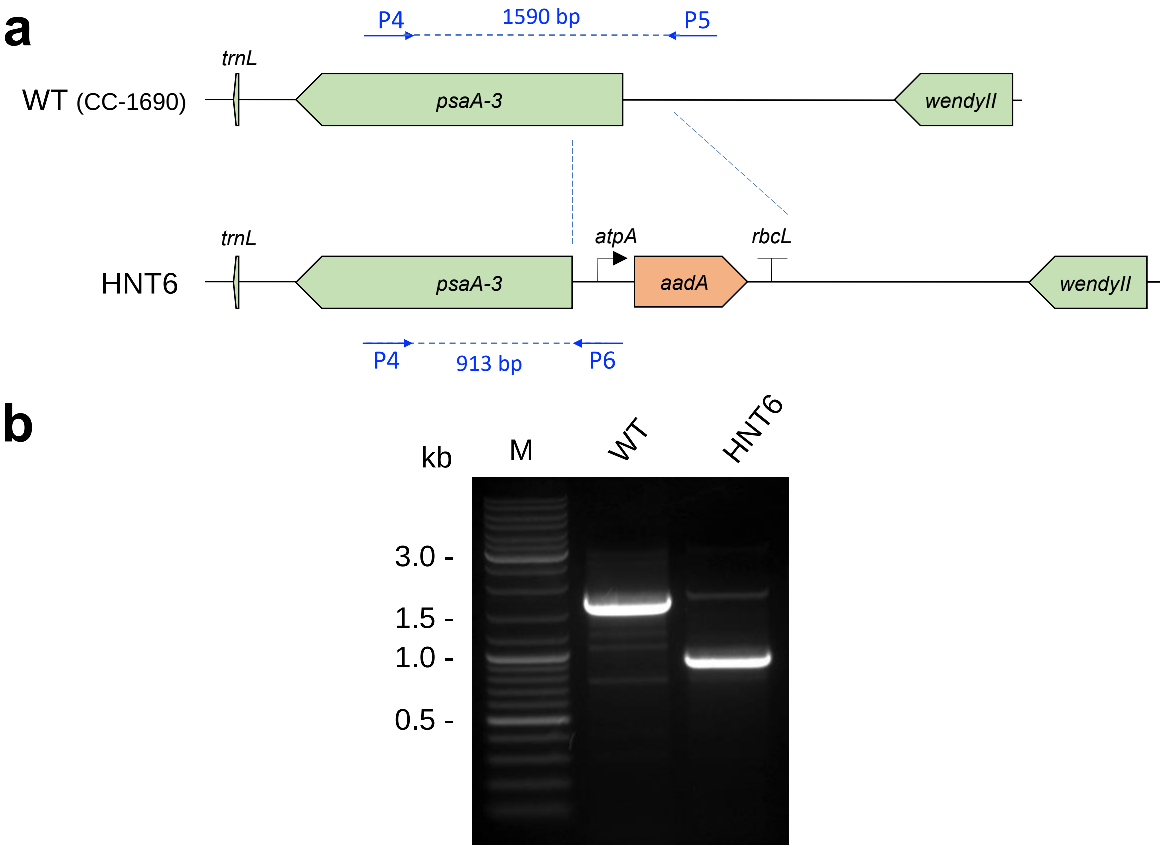

Figure S3. PCR analysis confirming homoplasmy of a selected HT161 transformant (renamed HNT6). Total genomic DNA isolated from the untransformed wild-type (WT) strain CC-1690 and from HNT6 was used for PCR analysis of the *psaA-3* locus. A three-primer reaction allowed for discrimination of the two alleles with primers 1+2 specific for the WT allele and giving a PCR product of 1590 bp, and primers 1+3 specific for the *aadA*-disrupted allele and giving a product of 913 bp. The absence of a WT band in the HNT6 analysis indicates that the strain is homoplasmic (*i.e.* contains only transformed plastome copies within its chloroplast).

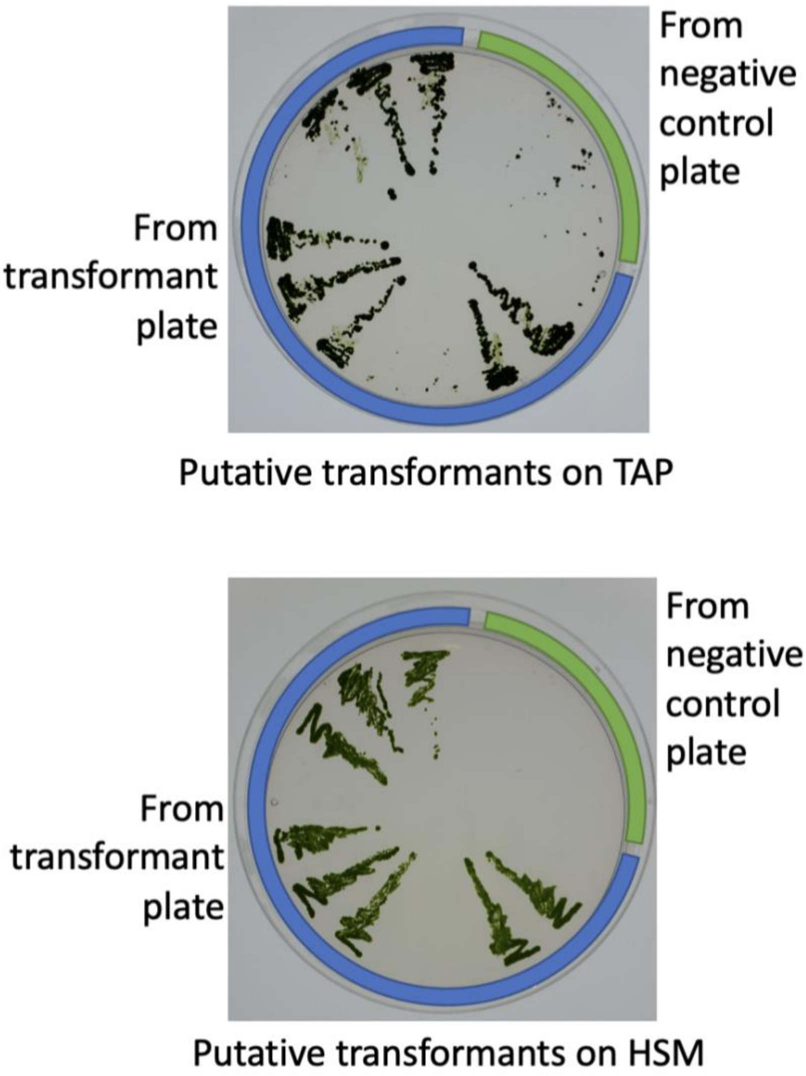

Figure S4. Growth analysis of 'true' transformants and 'false-positive' clones. Restreaking shows that true transformants of HNT6 grow well on TAP (acetate-containing medium) when cultured in the light, unlike false-positive clones. Twelve putative transformant colonies were restreaked following high-light selection on TAP medium, together with four colonies from a negative control TAP plate (see Figure 3). Those that grew well on TAP (upper panel) correlated with restoration of photosynthesis as seen on the HSM plates (lower panel).

Table S1. DNA primers used in the study

Primer name	Purpose	Sequence (5'–3')
HT247_5'_psaA_F	PCR for homoplasmy confirmation of HT158 and DNA sequencing (P1 in Fig. S2 above)	CCTGCTCGTGCCATAAATAAAAA
HT248_5'_psaA_R	PCR for homoplasmy confirmation of HT158 (P2 in Fig. S2 above)	AGTGCTTCCGCAACAAAAACTTC
QM125_PatpA_R	PCR for homoplasmy confirmation of HT158 (P3 in Fig. S2 above)	GGTCCTTTAAAGGTTTTATGGAC
Flank_F	PCR for homplasmy confirmation of HNT6 (P4 in Fig. S3 above)	AGTTATTAGTAGGATCGTAGTCACG
d/s_psaA-3_R	PCR for homplasmy confirmation of HNT6 (P5 in Fig. S3 above)	GTATATACATTCACCCTTTAAGGCTACC
atpA_R	PCR for homplasmy confirmation of HNT6 (P6 in Fig. S3 above)	CGTTTATGGTATATAAACACTGCC
16S_R	PCR for confirmation of homoplasmy	CCTTCCCCTTACGGGACAA
rbcL_R	DNA sequencing	GTTTAGGTATTTAACCTAAACAC
